# Technologies for participatory medicine and health promotion in the elderly population

**DOI:** 10.1097/MD.0000000000010791

**Published:** 2018-05-18

**Authors:** Laura Nieto-Riveiro, Betania Groba, M. Carmen Miranda, Patricia Concheiro, Alejandro Pazos, Thais Pousada, Javier Pereira

**Affiliations:** a Faculty of Health Science; b Faculty of Informatics, RNASA-IMEDIR Research Group, Universidade da Coruña, Spain.

**Keywords:** elderly, insomnia, quality of life, risk of falls, urinary incontinence, wearables

## Abstract

**Introduction:**

The progressive aging of the population is a socio-demographic phenomenon experienced by most countries in the world in recent decades, especially in Japan and in many European Union countries. During this process, so-called “geriatric syndromes” frequently occur. The focus of this study is the quality of life of the elderly in relation to these 3 factors: risk of falls, urinary incontinence, and insomnia.

**Objective:**

The main purpose is to determine the impact of a multifactorial intervention program implemented with institutionalized elderly people. The program is focused on the treatment of the aforementioned factors.

**Methods and Analysis:**

The study will be carried out with elderly people living in three residences for the elderly in A Coruña Province (Galicia, Spain).It is a prospective and longitudinal study, with a temporary series design of a “quasi-experimental” type that evaluates the effect of an intervention in 1 given population by doing assessments pre- and post-intervention, but there is no comparison with a control group.

The intervention will be based on a multifactorial program, including the following phases: the use of wearable devices (wearable fitness trackers to register physical activity and sleep), the use of an App on a Tablet to record the participants’ occupations and activities, counseling about performance in activities of daily living, the implementation of a physical activity program, and the treatment of the pelvic floor (according to each research line). The Quality of Life (QoL) will be assessed before and after the intervention, with the use of the questionnaire EuroQol-5D-5L. Data analysis will be applied with all registered variables through a quantitative perspective.

**Ethics and Dissemination:**

The protocol has been approved by the host institution's ethics committee (Research Ethics Committee of Galicia) under the number 2017/106. Results will be disseminated via peer-reviewed journal articles and conferences. This clinical trial is registered at ClinicalTrials.gov identifier: NCT03504813.

## 1 Introduction

### 1.1 Background and rationale

The progressive aging of the population is a demographic phenomenon in most countries of the world in recent decades, especially Japan and the countries of the European Union (EU).^[1]^ Today, people aged 60 years or older represent 23.8% of the total Spanish population, and those aged 65 years or older represent 18.4%. Thus, Galicia is among the greatest aging regions in Spain, both in absolute and relative numbers.^[2]^

Although aging is not a pathology or disease, it is known that old age is the life stage where the greatest risks exist for the appearance of pathology and/or chronic diseases. The World Health Organization (WHO) plans to detect and treat these diseases in time, in order to minimize their consequences, through a comprehensive primary care system.^[3]^

Characteristic of aging are the “geriatric syndromes,” and among them, the most frequent are:

(i) Urinary incontinence: the involuntary loss of urine through the urethra, objectively demonstrable and constituting for the person who suffers it a social and hygienic problem.^[4]^
(ii) Falls: involuntary events that cause people to lose balance and find themselves on the ground or other firm surfaces.^[5]^ The factor of falls can be intrinsic (related to the person) or extrinsic (derived from the activity or environment of the individual).
(iii) Insomnia: a condition characterized by an unsatisfactory amount or quality of sleep which persists for a considerable period. This disorder includes difficulties for the falling and/or staying asleep and early awakening in the final phase of sleep.^[6]^

These geriatric syndromes constitute the 3 research lines of the present project. Related to these syndromes are a wide range of possibilities for primary, secondary, and tertiary prevention, the ability to direct efforts to avoid or reduce the appearance of these disorders, improve early diagnosis and slow down its evolution, or reduce its complications and side effects.^[7–9]^

Relating the mentioned geriatric syndromes with the concept of “Internet of things” and the possibilities of new technologies, this project intends to integrate different sensor devices (or wearables) able to monitor users in a non-invasive way and feed the system with the collected data in order to offer specific interventions for different user profiles.^[10]^

Currently there are numerous commercial solutions for monitoring health parameters of users through sensor devices capable of transmitting data to other devices, such as a mobile phone or a computer. Among these solutions are quantifying bracelets for physical exercise and/or sleep, scales, glucometers, tensiometers, heart rate monitors, ans so on.^[11]^. However, the 3 research lines that we are considering in this study are neither adequately nor completely covered today by commercial devices. Therefore, a significant part of the research work consists of being able to define, based on the requirements indicated by the participating health professionals, a set of “models” of sensors capable of measuring the pertinent parameters, which is not possible by using only commercial solutions. On the other hand, the personal and non-transferable use that is going to be made of the sensors by the users forces the costs of the same to be moderate, which limits the range of devices that can be included. What matters, in short, are reliable and affordable sensor devices for the end user, without neglecting technology and efficiency.^[10]^

Participatory Medicine is a model of health care that highlights the active role of the patient, based on the collaboration and empowerment of the patient. Moreover, digital revolution allows for empowerment of patients, which helps them in their own treatment and care. Current studies are demonstrating that this type of responsibility from the patient significantly improves treatment times, reduces the time and amount of drug use and, importantly, causes people to increase their level of well-being by feeling they are part of the process of healthcare.^[12]^

The present research focuses on the study of the use of information and communication technologies by elderly people to promote their health. The objective of the project is to investigate and develop a technological solution that offers services oriented toward evaluation and intervention with the elderly in the 3 research lines: increasing urinary continence, detection, and prevention of falls and sleep control. However, the project will be designed and developed on a web-based-platform (transversal) to include other areas related to the elderly's health.

### 1.2 Objectives

The main purpose is to determine the impact on quality of life of a multifactorial intervention program implemented with institutionalized elderly people with urinary incontinence, sleep disorders, and/or risk of falls.

The specific objectives are:

(i) To facilitate the reduction of the symptoms and signs of these geriatric syndromes (urinary incontinence, sleep disorders, and risk of falls).
(ii) To analyze the changes produced after the intervention in the occupations of the elderly.
(iii) To promote the use of technology devices in the daily lives of the elderly, especially for the empowerment and management of their health.
(iv) To increase the responsibility and active participation of the elderly in their health and aging process.

### 1.3 Study design

The study will use a temporary series design of a “quasi-experimental” type, aimed at assessing the effect of an intervention on a given population by performing pre- and post-intervention measurements, but without existing comparison with a control group. The design is longitudinal and prospective.

This study protocol follows the Standard Protocol Items for Randomized Trials (SPIRIT).^[13]^

## 2 Methods: participants, interventions, and outcomes

### 2.1 Study setting

The study will be carried out in 3 residences for the elderly in 3 different cities of the A Coruña region (Spain). The project has duration of 36 months, from March 2017 to December 2019.

### 2.2 Eligibility criteria

There will be a convenience sample. Participants will be people older than 65 years old, living in 1 of the 3 residences involved in the study, who meet the inclusion criteria.

The general inclusion criteria is people with 65 years of age or older.

Specific criteria for each research line:

∘ Urinary incontinence:
  - To have stress, urgency or mixed urinary incontinence
  - To be a woman
∘ Insomnia:
  - Diagnosis of insomnia and/or hypersomnia.
∘ Risk of falls:
  - To have a previous history of falls in the last 6–12 months.
  - To present risk of falling and/or fear of falling.
  - To have independence in locomotion.

The general exclusion criteria are:

(i) Showing cognitive deterioration from moderate to very severe (Mini-Examination Cognitive <20 points).
(ii) Having severe, acute complications in health that prevent assiduity in attending interventions.
(iii) Diagnosis of conditions and/or pathologies in which physical activity is contraindicated (mainly cardiorespiratory diseases).
(iv) Being in the final stage of a terminal illness.
(v) Bing in a situation of request for transfer to another center.
(vi) Having a temporary stay in elderly residence.
(vii) Having a situation of legal incapacity.

Specifically, for the research line on urinary incontinence, several specific exclusion criteria have been established:

(i) Having functional urinary incontinence because that type is related to cognitive deterioration, urinary infection, polypharmacy, psychological problems, endocrinopathy, mobility restriction, and fecal incontinence.^[14]^
(ii) Having undergone surgery in the pelvic floor area.
(iii) Uterine prolapse, cystocele and/or rectocele (levels 3–4).
(iv) No control of the pelvic floor.

### 2.3 Interventions

**Figure 1:**
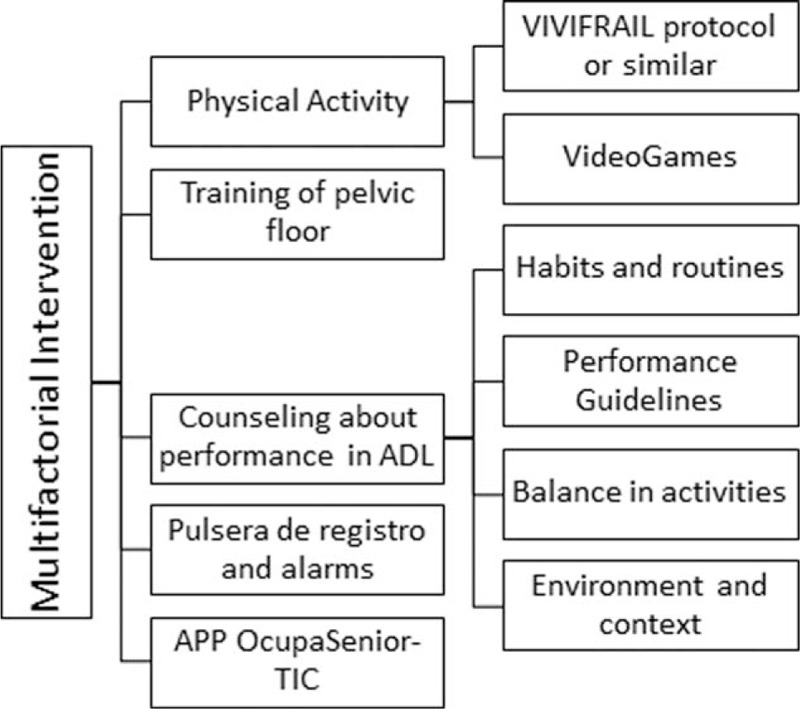
Scheme of Multifactorial Intervention Program.

Intervention will consist of a multifactorial program with the follow stages: the use of wearable devices (wearable fitness tracker to register physical activity and sleep), the use of an App on a Tablet to record the participants’ occupations and activities, counseling about performance in the activities of daily living, the implementation of a physical activity program, and the treatment of the pelvic floor (according to each research line). The process of intervention is showed in Figure 1.

From the beginning, participants will receive wearable fitness trackers to register data about physical activity and quality of sleep. The wearable device offers the possibility of programming alarms or notifications.

Moreover, participants will record information themselves, daily or monthly (according to type of data), about different aspects of their occupational performance. That register will be done through the App OcupaSenior-TIC. The Tablet with a specific App and the wearable device will be used transversally throughout the whole project.

The first sessions of intervention will be focused on training with the technological devices (Tablet and wearable). The number of sessions and the duration of training will depend on the needs of participants.

Once the different technological devices are integrated, the development of activities in the multifactorial intervention program will start: counseling about occupational performance, a physical activity program and relaxation or training of the pelvic floor, according to the research line in which participants are enrolled:

(1) Physical activity programs: To implement this line, the protocol of the VIVIFRAIL Project will be used as a reference. That protocol has different physical exercises, divided into itineraries, in order to adapt it to a person's capability.^[15]^ Several sessions of this research line will employ the use of videogames so that the elderly can explore new forms of physical activity and thus establish contact and expertise with new technologies. It is estimated that 3 sessions per week over 2 months are needed for each person to continue doing physical activity independently and with autonomy.
(2) The relaxation training will take place with those participants who are included in the research lines of insomnia and risk of falls. It is estimated that 2 sessions per week for 1 month are needed.
(3) The training of the pelvic floor will apply to participants enrolled in the research line dealing with urinary incontinence during the 6 months of intervention, with a frequency of 2 sessions per week.
(4) Counseling about occupational performance: After the physical activity and relaxation programs, the counseling will start. With each participant, routines and daily activities will be planned that will be adequate according to different recommendations about urinary incontinence, insomnia, and risk of falls. Different performance guidelines will be established in order to establish a good balance between activities. The guidelines can include the recommendations about adjustments to the environment. It is estimated that three sessions per week for 2 months are needed to incorporate advice and routines into the daily lives of participants.

All sessions will carry on in groups of 6 people, and their duration will be 45 minutes each.

### 2.4 Software

An App for Tablet with Android System will be designed and created, and it is called App OcupaSenior-TIC. This App will be a health manager, promoting participative health, in which participants will record, daily and monthly, their different occupations and/or relevant information concerning urinary incontinence, insomnia, and falls. This App will be linked with ClepIO, which is an online health application, to manage the clinical history and personal record of each participant's own health, as well as to monitor the pharmacological treatment.

### 2.5 Participant timeline

The first contact with possible participants started in April 2017. They were given the information letter and the informed consent at the same time. The process of assessment is periodic, starting in May 2017 and finishing in November 2018.

The intervention with each group of participants starts in July 2018, with a duration of 24 months. The final assessment will start in February 2019, 3 months after the end of interventions.

The complete timeline on development of the project is shown in Table 1.

**Table 1:**
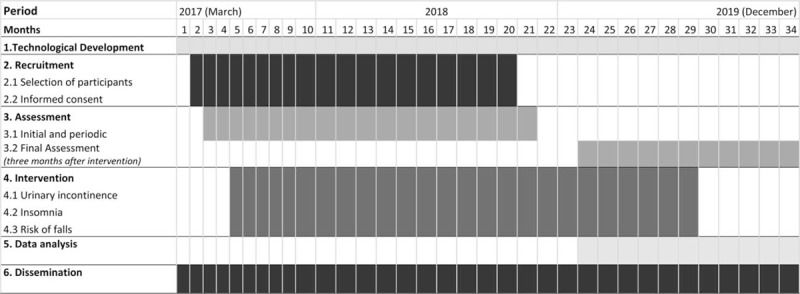
Timeline of research project.

### 2.6 Sample size

In order to get 95% safety, a power of 80%, a mean difference of 0.05, and a correlation coefficient of 90%, a sample of 57 people is needed. The size of the sample was calculated in order to obtain statistically significant results in relation to the main variable, which is the quality of life.

### 2.7 Recruitment

The contact with possible participants and their recruitment for the study will be done through specific calls in the residences, requesting the collaboration of the users, and reference professionals.

Subsequently, presentation of the project to possible participants in the facilities of the residences will be carried out. All assistants will receive an information letter about concerns and performance of the research study.

In order to formalize their collaboration, the informed consent procedure will be carried out with participants who meet the inclusion criteria.

## 3 Methods: data collection, management, and analysis

The main variable assessed in the present study is the quality of life. Additionally, research group has been identified both general, common variables for the 3 research lines, and specific variables for each of the 3.

### 3.1 Data collection methods

The quality of life (QoL), the main study variable, will be determined with EuroQol-5D-5L. This descriptive system comprises 5 dimensions: mobility, self-care, usual activities, pain/discomfort, and anxiety/depression. Each dimension has 5 levels: no problems, slight problems, moderate problems, severe problems, and extreme problems.^[16]^ The EQ Visual Analog Scale (EQ VAS) records the patient's self-rated health on a vertical visual analogue scale. This can be used as a quantitative measure of health outcome that reflects the patient's own judgment. The scores on these 5 dimensions can be presented as a health profile or can be converted to a single summary index number (utility) reflecting preferability compared to other health profiles.^[17]^ This tool is used in initial, periodic, and final assessments during the research project.

To get a general profile and complete the information about participants, a specific registry sheet was designed. On this sheet, sociodemographic and health data of the participants that may influence their quality of life are recorded: age, sex, marital status, educational level, main work activity, type of retirement, socioeconomic level, living environment, social support, use of assistive technology, diagnosis, and medication. This information will be consulted in the database of each residence or will be discussed with the elderly person, their relatives, or professionals.

The other general variables will be collected with the following methods:

- The Geriatric International Classification Functioning (ICF) Core Set: A comprehensive and valid set of 29 ICF categories, reflecting the most relevant health-related problems among community-living older adults. This consists of 35 items or categories which assess different conditions of health status. Each category is scored as 0, 1, 2, 3, 4, 8, or 9, except for the value of environmental factors, scoring on a scale as + 4, + 3, + 2, + 1, 0, 1, 2, 3, 4, 8, or 9. Environmental factors are scored as facilitators, barriers, or neutral elements.^[18]^
- Barthel Activities of Daily Living (ADL) Index: This tool has been used as a simple index of independence to score the ability of a patient to care for himself and by repeating the test periodically, to assess his improvement. The performance is assessed based on these scores: <20: total dependence, 20 to 40: severe dependence, 45 to 55: moderate dependence, and 60 or more: mild dependence. This tool is used in initial, periodic, and final assessments.^[19]^
- Tinetti Assessment Tool: A simple, easily administered test that measures a person's gait and balance. The test is scored on the individual's ability to perform specific tasks. “Scoring of the Tinetti Assessment Tool is done on a 3-point ordinal scale with a range of 0 to 2. A score of 0 represents the most impairment, while a score of 2 represents independence. The individual scores are then combined to form 3 measures; an overall gait assessment score, an overall balance assessment score, and a combined gait and balance score.” This tool is used in initial, periodic, and final assessments.^[20]^
‐ Mini-Mental State Examination (MMSE): This tool has been designed to determine the “cognitive state,” that is one of the study's variables. It will be used for screening in order to verify 1 of the established exclusion criteria and in the initial, final, and follow-up evaluations. It presents 6 values: one for each section (Orientation, Fixation, Concentration and calculation, Memory and Language, and Construction) and final score.^[21]^
- Charlson Comorbidity Index (CCI): Predicts 10-year survival in patients with multiple comorbidities.^[22]^

To get data with respect to patient-specific characteristics in each 1 of the 3 research lines, additional instruments have been selected:

- Urinary incontinence:
  - Oxford Grading Scale: This scale measures the variable of “pelvic floor contractile capacity” and will be used for screening since a person who does not have contractile capacity cannot participate in the research line regarding urinary incontinence. It has a unique numerical value (0–5), that is the result from assessment of the contractile capacity of the pelvic floor muscles.^[23]^
  - Sandvick Severity Index: This test assesses the variable “urine leakage” and will be used for screening in order to assess the severity of urinary incontinence. It presents a unique numerical value and consists of 2 questions, one of them about the frequency with which a person has urine leakage (maximum score of 4 points) and another with reference to the amount of urine in the exhaust (maximum score of 3 points).^[24]^
  - IU4 Questionnaire: This tool helps to classify the type of urinary incontinence (UI). This scale has a unique qualitative value (4 different options). It is emphasized that question 1 identifies the effort of UI, while questions 2 and 3 identify the UI of urgency.^[25]^
  - 24-Hour Pad Test: This tool allows to measure the “amount of urine” and will be used in the initial, periodic, and final evaluations. This scale has three values (net weight of the napkin/diaper, weight with urine, and weight difference). The average of 3 days (one measurement per day) every 15 days will be carried out.^[26]^
  - PERFECT Scheme: The study variable that allows assessment of the “pelvic floor musculature” and will be used in the initial, periodic, and final evaluations. PERFECT is an acronym with P representing power (or pressure, a measure of strength using a manometric perineometer), E = endurance, R = repetitions, F = fast contractions, and finally ECT = every contraction timed. The scheme was developed to simplify and clarify PFM assessment.^[27]^
  - International Consultation on Incontinence Questionnaire (Short Form): The ICIQ-SF is a self-administered questionnaire that qualifies the symptoms and quality of life in both male and female adult patients with symptoms of urine loss.^[28]^
- Insomnia:
  - Oviedo Sleep Questionnaire: This scale consists of 13 items with 3 values: one for each section (sleep satisfaction, insomnia and hypersomnia). Each item is scored from 1 to 5, except for item 1 which is scored from 1 to 7. The subscale of insomnia ranges from 9 to 45 points; the higher the score the greater the severity.^[29]^
  - Pittsburgh Sleep Quality Assessment (PSQI): This is a self-report questionnaire that assesses sleep quality over a 1-month time interval. The measure consists of 19 individual items, creating seven components that produce 1 global score and takes 5 to 10 minutes to complete. The PSQI is intended to be a standardized sleep questionnaire for clinicians and researchers to use with ease and is used for multiple populations.^[30]^
- Risk of falls:
  - Timed Get Up and Go Test: A simple test used to assess a person's mobility and requires both static and dynamic balance. It uses the time that a person takes to rise from a chair, walk three meters, turn around, walk back to the chair, and sit down. During the test, the person is expected to wear their regular footwear and use any mobility aids that they would normally require.^[31]^
  - Falls Efficacy Scale International (FES-I): A short, easy to administer tool that measures the level of concern about falling during social and physical activities inside and outside the home whether or not the person actually does the activity. The level of concern is measured on a 4-point Likert scale (1 = not at all concerned to 4 = very concerned).^[32]^

### 3.2 Data management

To measure the efficacy of the multifactorial program, an initial assessment will be carried out before the intervention, in combination with periodic or follow-up evaluations. A final assessment will take place after the intervention and another one in 3 months after the end of the intervention. The tools used in the study, according to general research and specific research lines, are:

- General tools: EQ-5D-5L, CIF Basic set of Abbreviated Geriatric Patients, MEC, Barthel Index, and Tinetti Scale.
- Urinary incontinence: ICIQ-SF, PERFECT, and 24-Hour Pad Test.
- Insomnia: PSQI.
- Fall risk: Tinetti scale, Timed Get Up and Go, and FESI.

The confidentiality of all the data collected and the anonymity of each participant will be maintained. The data of the participants will be collected and preserved until the end of the study in coded mode. To do this, each participant will be assigned an alphanumeric code consisting of the letter P and a correlative number.

### 3.3 Statistical methods

The analysis of the data will be done with the statistical program SPSS. The quantitative variables will be expressed with the mean and the standard deviation, while the qualitative variables will be expressed as an absolute value and percentage.^[33]^

To compare the means, the student's *t* test will be used, and for the multiple comparisons of means, the analysis of the variance will be used. This test allows us to determine if the differences between the values of both variables are statistically significant or if they are differences due to chance. To study the association between qualitative variables, the *χ*^*2*^ test will be used.

On the other hand, to determine the variables that are associated or not with the presence of the dichotomous variable of interest (Quality of Life), a multivariate logistic regression analysis will be performed, using as a dependent variable the presence or lack of presence of the event of interest, and as covariables, the variables that in the bivariate analysis are associated with the presence of said event or are clinically relevant.^[33]^

## 4 Ethics and dissemination

### 4.1 Research ethics approval

This study protocol has been approved by the host institution's ethics committee (Research Ethics Committee of Galicia) under the number 2017/106, with the date of ^t^ March 21, 2017.

The protocol is registered in ClinicalTrials.gov, with the reference number: NCT03504813

### 4.2 Protocol amendments

To communicate important possible amendments introduced in the protocol, a new request to the Research Ethics Committee of Galicia will be done, with the reference number assigned. Research group will wait for approval from this ethics committee in order to continue with the study. These amendments will also be updated in the registry of ClinicalTrials.

### 4.3 Consent or assent

With each participant, the process of informed consent will be applied. Participants will receive complete verbal and written information about characteristics of study and about the implications derived from their participation in it. The Information Sheet will be given to each participant so they can read it slowly and take the needed time to ask all questions that they have. Once it has been ensured that all participants fully understand the information provided, they will accept if they wish to participate in the study through the Informed Consent Document.

### 4.4 Confidentiality

The main researcher maintains the confidentiality of all data collected and the anonymity of each participant. Thus, the Spanish Organic Law on the protection of personal data will be respected at all times.

The data of the participants will be collected and preserved until the end of the study in coded mode.

### 4.5 Declaration of interests

The authors declare that the research will be conducted in the absence of any commercial or financial relationships that could be construed as a potential conflict of interest.

### 4.6 Access to data

The custody of documentation that relates the identity of the participants with the coding will be the responsibility of the collaborating researchers. In this way, it is guaranteed that the rest of the researchers cannot know the identity of the participants at any time during the investigation. At the end of the project, the data will be anonymized.

### 4.7 Dissemination policy

Once the results and conclusions of the study have been extracted, they will be disseminated through the publication of scientific articles in international journals with high impact. The main investigator undertakes the publication of the results obtained, both negative and positive, and guarantees the anonymity of said data at all times.

## 5 Discussion

The development of this project represents an important innovation in the current scenario of patient-monitoring systems based on the Personal Health Record paradigm. The integration of characteristics of this paradigm in a single platform that allows, autonomously and freely, sharing and managing health information among patients, health professionals, and families is an innovative approach in European countries.

Other considerations that the project incorporates into this research field are:

- The development of intervention protocols for three areas of special concern for the elderly: urinary incontinence, the existence of falls, and insomnia.
- The conduction of a study which, by involving and empowering the elderly person in the control of their health, allows greater autonomy in this vital stage, decreasing continued external support.
- The adaptation of an App and interfaces for the elderly user who has some type of disability (cognitive, sensory, and/or physical).
- The integration of an App and wearables to verify, in real time, the execution of the proposed activities as an intervention.

In order to obtain more advances in this field and to complement the results derived from this study, we propose future lines of research:

- Clinical research: the important volume of health data that will be registered in the database will allow the information to be exploited to try to generate new knowledge. Applying data mining and big data techniques, it will be possible to obtain trend information and search for correlations between data, providing a basis for addressing research projects in health.
- Expanding the 3 research lines to other relevant areas in the aging of the population, for example, the prevention of cardio-respiratory problems, hearing loss, and so on.
- Conducting a clinical trial.
- Integrating into the solution wearable devices that come onto the market or devices of different types and/or brands, both in the national and international market and analyzing the data patterns in comparison with the patterns obtained in this project.

## Author contributions

**Conceptualization:** Laura Nieto-Riveiro, Betania Groba, Carmen Miranda, Patricia Concheiro, Thais Pousada, Javier Pereira.

**Formal analysis:** Laura Nieto-Riveiro, Betania Groba.

**Funding acquisition:** Alejandro Pazos, Javier Pereira.

**Investigation:** Laura Nieto-Riveiro, Betania Groba, Carmen Miranda, Patricia Concheiro.

**Methodology:** Laura Nieto-Riveiro, Betania Groba, Carmen Miranda, Patricia Concheiro.

**Project administration:** Laura Nieto-Riveiro, Javier Pereira.

**Supervision:** Alejandro Pazos, Javier Pereira.

**Validation:** Laura Nieto-Riveiro, Betania Groba, Alejandro Pazos, Javier Pereira.

**Visualization:** Betania Groba.

**Writing – original draft:** Thais Pousada.

**Writing – review & editing:** Thais Pousada.
